# Are friends electric? The benefits and risks of human-robot relationships

**DOI:** 10.1016/j.isci.2020.101993

**Published:** 2020-12-26

**Authors:** Tony J. Prescott, Julie M. Robillard

**Affiliations:** 1 Department of Computer Science, University of Sheffield, Sheffield, UK; 2 University of British Columbia, Vancouver, BC, Canada

**Keywords:** Cognitive Neuroscience, Psychology, Research Methodology Social Sciences

## Abstract

Social robots that can interact and communicate with people are growing in popularity for use at home and in customer-service, education, and healthcare settings. Although growing evidence suggests that co-operative and emotionally aligned social robots could benefit users across the lifespan, controversy continues about the ethical implications of these devices and their potential harms. In this perspective, we explore this balance between benefit and risk through the lens of human-robot relationships. We review the definitions and purposes of social robots, explore their philosophical and psychological status, and relate research on human-human and human-animal relationships to the emerging literature on human-robot relationships. Advocating a relational rather than essentialist view, we consider the balance of benefits and harms that can arise from different types of relationship with social robots and conclude by considering the role of researchers in understanding the ethical and societal impacts of social robotics.

## Graphical abstract

**Figure:**
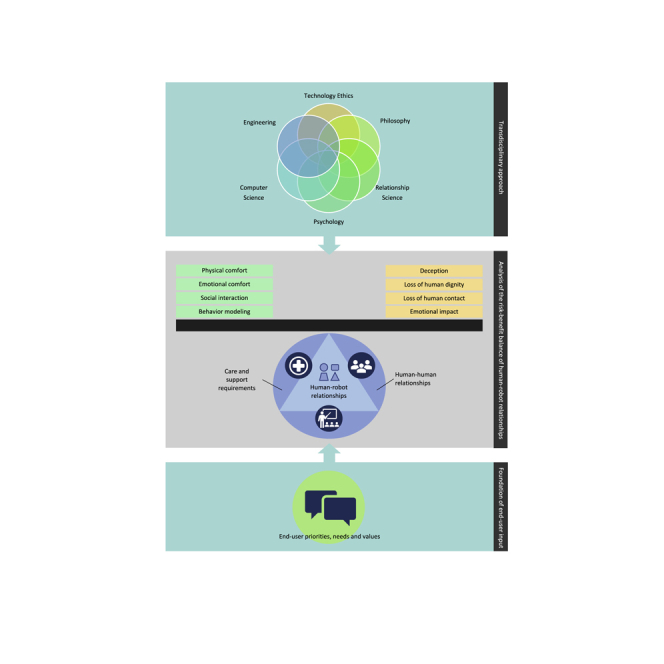

## Introduction

**Figure 1:**
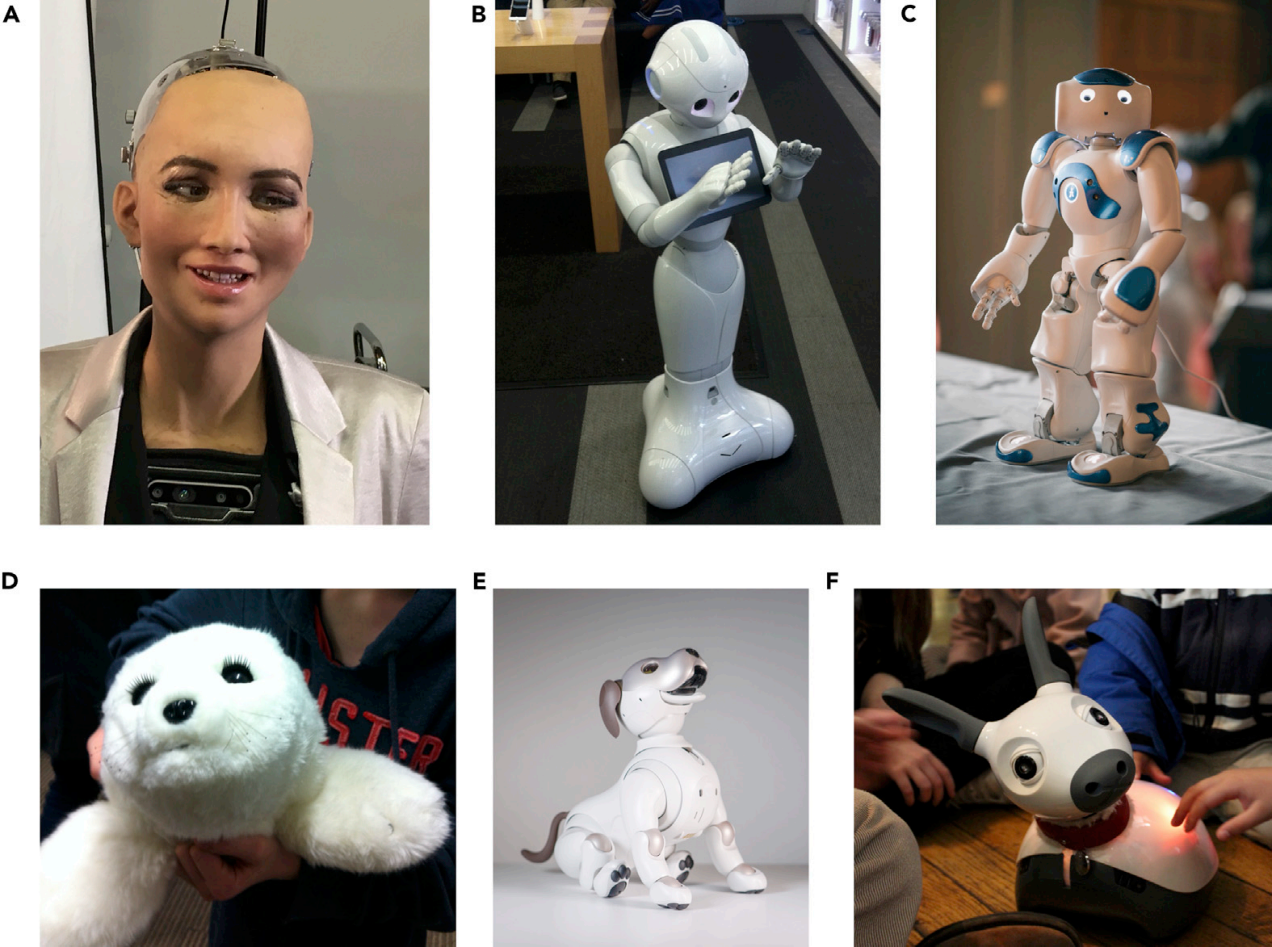
Examples of humanoid and animaloid social robots (A) Sophia (Hanson Robotics), (B) Nao (Softbank), (C) Pepper (Softbank), (D) Paro (Paro Robotics), (E) Aibo fourth generation (Sony Corporation), (F) MiRo-e (Consequential Robotics). Credits: (A, B, D, and F) Tony Prescott; (C) The University of Sheffield; (E) Paul Killeen.

Social robots, defined as robots that interact and communicate with humans or other agents by exhibiting social behaviors and following norms, have exploded in popularity in recent years, with a rapid growth in the development of research prototypes and in the commercialization of devices. Well-known examples of social robots, illustrated in Figure 1, include Hanson Robotics' *Sophia*, a human-like robot that captured the media's attention in 2017 when it was granted “honorary citizen” status in Saudi Arabia (Pagallo, 2018), and Softbank's *Pepper* (Pandey and Gelin, 2018) and *Nao* (Gouaillier et al., 2009), two widely available humanoid robots used in research across various disciplines and for commercial applications in customer assistance and education. Non-humanoid robots include animal-like solutions such as Sony's playful robotic dog *Aibo* (Fujita, 2000) (redesigned and relaunched in 2018), the seal-like *Paro*, developed to have a calming effect on residents of long-term care facilities (Shibata and Wada, 2011), and *Miro-e,* a bioinspired robot developed for applications in education and therapy (Prescott et al., 2018).

The interactive and dynamic features of social robots, and their ability to understand and respond to human emotion, make them candidate solutions for application domains that require social engagement and comfort. In healthcare, social robots are being investigated and implemented as tools to assist patients by means of emotional support across the lifespan, from pediatric populations (Kabacińska et al., 2020) to older adults (Broekens et al., 2009; Robinson et al., 2014; Prescott and Caleb-Solly, 2017; Pu et al., 2018; Papadopoulos et al., 2020). In education, social robots are being used as tutors or co-learners to promote cognitive and affective outcomes including social skills development (Belpaeme et al., 2018). A particular focus has been on populations that experience challenges with sociability and attention, such as children with autism spectrum disorder (ASD) (Cabibihan et al., 2013; Pennisi et al., 2016). There is growing use of social robots as home companions as alternatives to animal pets, where they have been found to have a positive impact on the experience of loneliness (Kanamori et al., 2002; Banks et al., 2008). Some of these application areas have raised concern, for example, in situations where young children, or older adults living with cognitive impairment, were confused as to whether a social robot was a living entity (Sharkey and Sharkey, 2012a), or in situations where people have appeared to place undue trust in a guide robot (Robinette et al., 2016). The emerging use of anthropomorphic robots as sex companions has also generated significant controversy (Levy, 2007; Richardson, 2016; Bartneck and McMullen, 2018; Döring et al., 2020). Although the use of robots in most of these settings has barely begun, the debate about the ethical risks they raise is already in full swing and is frequently featured in mainstream media.

In this perspective, we review social robots through the lens of human-robot relationships. We examine commonalities and differences between our relationships with social robots and with a range of social others (humans, animals, objects) and discuss ways in which human-robot bonds could benefit or harm individual end users and society as a whole. We also identify some of the knowledge gaps that can be addressed to provide a strong evidence base for the future development of social robots that can promote their beneficial use and mitigate risks.

## What is a social robot?

Robots are physical machines that embed elements of computational intelligence that enable them to behave autonomously (Bekey et al., 2008), often with the ability to operate for long periods of time without direct human control or supervision.

A subset of robots are “social” or “socially assistive” (Matarić and Scassellati, 2016), in that they typically integrate some capacity for aural (e.g. spoken) and non-aural communication (Mavridis, 2015), often having a human or animal-like appearance (humanoid and animaloid, respectively) that presents familiar social cues to people, such as a face with clearly visible “eyes” (DiSalvo et al., 2002), and displays or actuators that support expression and gesture (Venture and Kulić, 2019).

In order to safely interact and communicate effectively with people, social robots must embed a control system, or “cognitive architecture” (Kotseruba et al., 2016), that includes capacities for aural and non-aural communication, scene analysis, person and object detection and recognition, world knowledge, memory, action, and interaction planning. Social robots differ substantially in the design and configuration of this architecture, many being directly inspired by human or animal psychology and neuroscience (Asada et al., 2001; Arkin et al., 2003; Lungarella et al., 2003; Verschure, 2012; Cangelosi et al., 2015; Mitchinson and Prescott, 2016; Cross et al., 2019).

Cognitive architectures may include internal models of the robot's physical morphology and of the nearby environment, tracking its current position and pose to enable safe planning of movement and human-robot physical interaction (e.g. Moulin-Frier et al., 2017; Metta and Cingolani, 2018). Cognitive architectures have also been devised that store memories of past events (e.g. Dominey et al., 2017; Prescott et al., 2019) and that construct models of others' actions, beliefs, desires, and intentions (e.g. Scassellati, 2002; Johnson and Demiris, 2005; Trafton et al., 2013; Devin and Alami, 2016; Moulin-Frier et al., 2018), thus providing some of the cognitive capacities, such as autobiographical memory and “theory of mind,” that underpin human social cognition.

Social robots must exchange with humans in a natural manner that is easily understood and follows contextually appropriate norms (Arkin and Moshkina, 2015; König et al., 2017; Robillard and Hoey, 2018). In addition to spoken language processing, some humanoids can display recognizable human-like facial expressions such as smiles or frowns (e.g. Breazeal, 2001; MacDorman and Ishiguro, 2006; Cameron et al., 2018), whereas movement, posture, sound, prosody, and color can also provide forms of affective communication (e.g. Breazeal, 2001; Arkin et al., 2003; Collins et al., 2015; Venture and Kulić, 2019; Ghafurian et al., 2020). As mood and emotion are integral components of human interaction, there have been significant efforts made toward the development of emotionally responsive robots (Arkin et al., 2003; Kirby et al., 2010; Calvo et al., 2015; Collins et al., 2015). By including model motivational components, the display of affective signals can be aligned to internal drive and reward systems and to the social context (e.g. Breazeal, 2001; Fischer et al., 2018). Several models of affect have been put forward that implement this mapping, including dimensional models (e.g. Arkin et al., 2003; Collins et al., 2015), affect control theory (Robillard and Hoey, 2018), categorical models (Kirby et al., 2010), and appraisal models (Marcello et al., 2012).

This state-of-the-art in cognitive architecture enables forms of social behavior that are beginning to approach the level of sophistication seen in simpler forms of dyadic human-human interaction and can provide for engaging interactions that last over a period of time. Nevertheless, there is an asymmetry between the capacity of current robots to generate spoken language and expressive behavior compared with their ability to grasp the situational context, follow and participate in natural dialogue, or read people's intentions. This stands in contrast with the trajectory of human development where social comprehension typically precedes production—a social robot can appear to talk like an adult and yet have less situational understanding than a 2-year-old. This imbalance, which can be confusing for users, will reduce as the context sensitivity of robot social intelligence improves. In the meantime, human-robot verbal interactions are most effective within constrained settings, with clearly defined interaction goals. Scaling back the production capabilities of social robots to better match their cognitive sophistication can also be an effective strategy. For instance, animaloid robots can serve in companionship or therapeutic roles while being largely non-verbal and with limited situational awareness. An animal-like appearance may also generate significantly less user expectation, regarding the robot's cognitive and social capacities, compared with humanoids.

Social robotics is able to leverage broader advances in artificial intelligence (AI) and robotics, including improvements in machine learning, computer perception, natural language processing, and robot control. Although current social robot platforms have limited onboard processing, they are also increasingly able to take advantage of cloud computing to support more intensive forms of computation; this will help deliver richer, and more context-aware, social interaction capabilities. Social robots are also able to access resources such as off-board sensors and databases that will allow them to provide forms of social support that are less easy and natural for people. Thus, rather than duplicating or emulating forms of human to human interaction, we can expect that relationships with robots will have somewhat different, and often complementary, qualities.

## Perceptions and concerns about social robots

On the whole, people are positively disposed toward social robots and interested to engage with them (Naneva et al., 2020). The development of social robotics has nevertheless generated significant concern leading to multiple efforts to characterize the ethical and societal challenges they raise (Feil-Seifer and Matarić, 2011; Lin et al., 2012; Torresen, 2018; Vandemeulebroucke et al., 2018). In response, there has been an international drive toward the development of design standards and guidelines (Winfield, 2019).

The physical nature of social robots, their exterior and behavioral resemblances to ourselves or other animals, and frequent (and often negative) representation in cultural artifacts such as film and literature (Payr, 2019) may contribute to feelings of unease, perhaps triggering anxieties about our own human nature, our relationship with the technologies we create, and their potentially dehumanizing influences (Kang, 2011; Szollosy, 2016). It is not surprising, then, that robots have become something of a poster child for societal concerns about the broader impacts of AI.

Much of the debate about the ethics of social robots revolves around questions of what robots *are* compared with how they are *seen* by people, in other words, around the contrast between their ontological and psychological status (Kahn et al., 2007; Coeckelbergh, 2010a, 2011; Prescott, 2017). Ontologically, social robots clearly belong to the class of designed machines; however, their ability to exhibit behaviors previously manifested only in living systems, and closely tied to human sociality and culture, places them near to category boundaries, thus challenging pre-existing definitions and distinctions (Kang, 2011; Prescott, 2017). Moreover, although we may consider that robots belong to the category of machines or tools, it appears that we often think and behave toward them as though they have psychological capacities more similar to ourselves and unlike conventional machines.

This ambiguity has led to suggestions that social robots should be seen as belonging to a new ontological class (de Graaf, 2016; Kahn et al., 2017).

Following the cognitive scientist Daniel Dennett (1987), we can classify our perceptions of entities such as social robots as belonging to different perspectives or “stances.” For instance, we may see them as *physical* objects that obey laws such as gravity (“if this robot tips past a certain point it will fall over”), as *designed* objects that have been engineered for particular purposes (“the face of this robot has been designed to emulate a human smile”), or as *intentional* agents that act rationally and in accordance with internal goals (“this robot is listening to me as it wants to be helpful”).

Research suggests that we see robots as intentional entities (Marchesi et al., 2019), as we do with other kinds of “social” machines (Reeves and Nass, 1996; Broadbent, 2017). However, this does not imply that we do not also see them as physical and designed. Cameron et al. (2017) found that children were more likely to rate the expressive social robot *Zeno* as “like a machine” when they saw the operator physically activate the robot, by pressing a button on its chest, than when the same behavior was triggered by a remote command. Thus, perception of autonomy may be an important factor alongside physical appearance and social behavior. More broadly, our prior expectations about robots appears to influence the extent to which we see them as intentional or machine-like (Perez-Osorio et al., 2019), suggesting that attitudes may change with time.

Robbins and Jack (2006) have added to Dennett's stances a *phenomenal* view, that is, seeing an entity as having some capacity for experiential awareness or as a moral agent. Interestingly, Huebner (2010) found that people were more willing to attribute intentional capacity to robots than any form of phenomenal capacity; thus, we may recognize robots as being similar to us in some ways but not in others. It is worth noting that such perceptions and attributions may also differ across cultures and can depend on values and world views (Kaplan, 2004).

## Varieties and dynamics of relationships

Humans have been described as ultrasocial animals (Tomasello, 2014)—we engage in many collective behaviors and belong to multiple nested and intersecting social groups and hierarchies. Social experiences are some of the primary drivers for feelings of pleasure, happiness, security, and self-esteem (Reis et al., 2000; Harris and Orth, 2019). The experience of social rejection, exclusion, or isolation can be traumatizing and can impact our physical health (Gulrez et al., 2015; Courtin and Knapp, 2017; Holt-Lunstad, 2018; Slavich, 2020); an absence of appropriate social contact, particularly early in life, can have long-lasting and damaging impacts (Bowlby, 1969). Nevertheless, our urge for sociality is not straightforward compared with other needs. Thirst leads us to seek water, hunger—calories, cold—insulation or heat. Our social drive, by contrast, is not about correcting an immediate homeostatic imbalance that, without action, would become a threat to life, although it has evolved to be closely tied to these core life processes (Slavich, 2020). Addressing social needs creates a life-setting in which humans are better able to thrive along multiple dimensions (Holt-Lunstad, 2018); this includes help and support not only to meet basic needs but also to achieve higher-level goals concerned with having a fulfilling and meaningful life.

We distinguish different categories of social relationships and typically look to different people to form these relationships with (Reis et al., 2000). If we are to consider the benefits and risks of human-robot relationships then we should examine how these might share similarities with, or impact upon, these different classes of human-human relationship.

Categories of human-human relationship include primary caregivers, relatives, long-term partners, lovers and sexual partners, friends, pen/online pals, colleagues, teachers, acquaintances, service providers (including carers, therapists, assistants, waiters), and celebrities, among others. Note that some relationships belong to multiple classes—for example, a service provider or colleague can become a friend (Sias and Cahill, 1998; Price and Arnould, 1999)—and that many, but not all, relationships are reciprocal (for instance, admiration for a celebrity is entirely one-way). Biological relatedness is a further factor besides level of intimacy, reciprocity, hierarchy, gender, identity, and group membership (Levinger, 1980; Bugental, 2000; Reis et al., 2000).

In addition to our relationships with each other, people can also have valued relationships with domestic animals (Schicktanz, 2006; Coeckelbergh, 2011; Borgi and Cirulli, 2016) and with objects (e.g. car) and cherished items (e.g. wedding rings) including where they provide forms of connection with human others or act as a proxy for others (Keefer et al., 2012; Collins et al., 2013).

That the variety of human-other relationships covers such a wide span is one of the reasons that the notion of relationship is itself weakly defined (Reis et al., 2000). Relationships arise when one individual or entity has an influence on another or where there is mutuality of influence. Human-human relationships also have temporal dynamics and emerge, are maintained (Oswald et al., 2004), or become dissolved, through cycles of exchange that can be reinforcing (where one positive social act is reciprocated with another), or destructive (such as the cycles of negative reciprocity seen when relationships break down) (Reis et al., 2000). The social expectations, norms, and schema, that structure everyday interactions, are also critical to the construction and management of relationships (Levinger, 1980; Holmes, 2000; Tomasello, 2014). Relationships exist within networks that can be nested or overlapping, and that change, or can have increased or reduced importance, across the lifespan (Nicolaisen and Thorsen, 2016). Processes at the community and society level impact on how relationships evolve and function, whereas perceptions of support help determine our experience of social connectedness (thus we can feel lonely in a crowd or together in solitude). The impacts of relationships with robots need to be analyzed and understood within this broad and complex social and psychological context (Reis et al., 2000; van Oost and Reed, 2011; Holt-Lunstad, 2018; Ostrowski et al., 2019).

**Figure 2:**
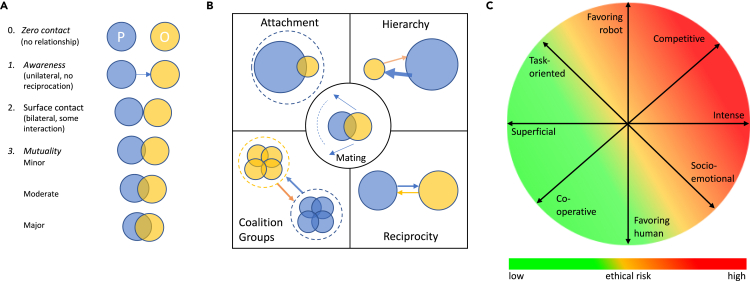
Ways of thinking about relationships (A) Levinger proposed four logically distinct levels of relatedness represented in terms of the degree of overlap between a person “P” and a social other “O.” The scale is intended to facilitate ways of measuring levels of mutual interdependence and the study of changes in depth of relationship over time (adapted from Levinger, 1980). (B) As part of a critique of unitary theories of relationships, Bugental (2000) developed a framework composed of five domains of socialization—attachment, hierarchy, coalitions, reciprocity, and mating—drawing on cognitive, developmental, evolutionary and social psychology, psychobiology, and behavioral ecology. These different domains are held to relate to different life challenges, to involve different psychological substrates, and to be regulated by distinct neurohormonal systems (redrawn from Bugental, 2000). (C) Wish et al. (1976) extracted a four-dimensional model of dyadic relationships from questionnaire data. Here we re-represent these dimensions in relation to human-robot relationships, highlighting the increased ethical risk of relationships that are more socioemotional, intense, competitive, or unequal (favoring the robot). See Reis et al. (2000), Bugental and Grusec (2006), and Holt-Lunstad (2018) for wider reviews of conceptual frameworks developed in human relationship science.

Human-human relationships have been variously characterized according to levels of relatedness or mutual interdependence (Levinger, 1980) and linked with different domains of social engagement tied to distinct bodies of social know-how and underpinned by diverse neuropsychological and developmental processes (Bugental, 2000; Bugental and Grusec, 2006). Using multidimensional scaling, Wish et al. (1976) analyzed ratings of twenty-five typical role relations in dyadic interactions identifying four principal dimensions interpreted as cooperative/friendly versus competitive/hostile, intense versus superficial, socioemotional/informal versus task-oriented/formal, and equal versus unequal. These different ways of thinking about relationships might be useful in considering human-robot relations with regard to both their similarities to human-human relationships and the level of ethical risk (see Figure 2). For example, a robot application such as customer assistance, could, according to Wish et al.’s dimensional model, be viewed as requiring a relationship that is cooperative, superficial, task-oriented, and favoring the human (for example in their ability to initiate or terminate an exchange). Such relationships potentially entail less ethical risk than those that are more intense, socioemotional, competitive, or have a power dynamic that is more equal or favors the robot in some way.

## Socioemotional relationships with robots

People can spontaneously form socioemotional bonds with robots, even those that are not specifically designed to elicit social behavior, as demonstrated by evidence of emotional attachment in owners of home-cleaning robots (Sung et al., 2007) and in soldiers working alongside bomb-disposal robots (Carpenter, 2015). In 2015, a Buddhist temple in Japan made world headlines by conducting a ceremony for *Aibo* robot dogs that were due to be dismantled (Brown, 2015). Priests in Japan routinely hold similar ceremonies for deceased pets; however, that such a ritual should be held for robots is indicative of significant attachment between owners and their robots.

The philosopher John Danaher has considered the nature of human-robot friendship, on the basis of the distinction, originated by Aristotle, and popular in many contemporary discussions of friendship, between “virtue” friends and “utility” and “pleasure” friends (Danaher, 2019). The virtue friend, according to Aristotle and Danaher, is one “that is premised on mutual good will and well-wishing, and that is pursued out of mutual admiration and shared values on both sides (Danaher, 2019, p. 9).” The utility friend, on the other hand, is one who helps you achieve some instrumental gain, and the pleasure-friendship is one where the principal benefit is a hedonic one. For Aristotle, the virtue friendship was “perfect” and other forms of friendship “imperfect.”

Discussion and refinement of the virtue friend concept has led to it be associated with the conditions of mutuality, authenticity, equality, and diversity of interaction (Danaher, 2019). For several commentators (e.g. de Graaf, 2016; Elder, 2016), robots could meet the weaker criteria for utility or pleasure friendship, but due to their differences from humans, and the inevitable asymmetries of any human-robot relationship, relationships with robots could never amount to a true (virtue) friendship. For Danaher, however, these challenges are technical rather than metaphysical. Specifically, he argues that if we assess friendship with robots against the same performative criteria by which we might judge relationships between people, and if we can design and build robots that meet these requirements, then the conditions for virtue friendship could be met.

It is unclear, however, whether the Aristotelian framework is the correct benchmark for evaluating human-robot relationships. The normative aspects of Aristotle seem particularly troubling, given the implication that departure from equality and shared values makes a friendship less than perfect. Many important human-human relationships, including those that involve care of vulnerable populations, are not easily characterized in these terms. The equality and mutuality conditions of virtue friendship are also not met in the case of human relationships with companion animals. Even so, based on other considerations, such as unconditionality and loyalty, human-animal relationships are widely held up as an alternative paragon of close friendship. That people find intrinsic value in different forms of asymmetric relationship suggests that this could also extend to robots.

Less widely noted in discussions of human-robot relationships, than the Aristotelian view, is the concept of friendship developed within Buddhism with its emphasis on the duties of friendship, including requirements to show generosity, to speak kindly, to act to improve the other's welfare, and to show reciprocity, impartiality, and honesty (Hruschka, 2010; Traud-Dubois, 2010). Although to behave in this way at all times can be challenging for humans, the capacity to act according to such considerations can be programmed into social robots.

Placing undue emphasis on ideals risks marginalizing the actual (Mills, 2005) and, in this case, excluding valuable forms of social contact. As Capiocco and Patrick (2008) have suggested, writing on the topic of human loneliness, “perfect friendships are impossible, but by reaching out beyond ourselves we can achieve the next best thing—social connection that is rich and satisfying.” For people who are socially isolated, this suggests that there is a *prima facie* case for exploring the potential well-being benefits of social connectedness with and through robots.

## Potential benefits

Across the lifespan, research suggests positive impacts of social robots across five overlapping dimensions: (1) physical comfort; (2) emotional comfort; (3) direct social interaction and scaffolding of social interactions with others, and (5) behavior modeling. Interventions, such as using social robots as therapeutic tools, could harness one or more of these dimensions, and longer-term relationships, such as the use of companion robots at home or in care, could also sustain multiple areas of benefit.

The role of social robots in providing physical comfort has been demonstrated in studies that compare interventions with social robots with controls using a tablet-based avatar of the same robot. Results consistently show greater engagement and more positive affect in the embodied intervention (Li, 2015), both in pediatric populations (Logan et al., 2019) and in older adults (Mann et al., 2015). As one example, in a randomized pilot trial, Logan et al. (2019) studied the responses of 54 children to one of three conditions: (1) a tele-operated bear robot, (2) an avatar version of the robot displayed on a tablet; and (3) a static plush bear. Children in the robot condition expressed greater joyfulness and agreeableness than those in the two other conditions. In a review of thirty-eight experimental studies comparing co-present robots, telepresent robots, and virtual agents, Li (2015) found that robots had greater influence on participants when physically present and elicited more favorable responses when compared with other agents. Barber et al. (2020) studied children’s free play with the animal-like robot Miro-e, comparing it with interactions with a living therapy dog. Children engaged in social touch with both the dog and the robot but, overall, spent more time interacting with the robot. Emerging work on affective touch in human-robot interaction also supports the value of physical contact with artificial companions in providing comfort (Flagg and MacLean, 2013; Sefidgar et al., 2016; Kerruish, 2017; Krichmar and Chou, 2018).

To the extent that robots can act as companions, they could plausibly act to reduce social isolation and the experience of loneliness (Gulrez et al., 2015). A study of the use of the Sony *Aibo* robot dog in a residential care home found a positive impact on the experience of loneliness similar to that generated by interaction with a real dog (Banks et al., 2008). Recent work on social robots as interventions for mental health also indicates the potential for the affective components of human-robot interaction to generate emotional comfort and to scaffold feelings of self-worth (Ostrowski et al., 2019; Kabacińska et al., 2020). The effectiveness of robots as social companions can also be improved by adapting their cognitive architectures and capabilities to suit specific populations, such as people living with dementia (Perugia et al., 2020).

By definition, social robots support communication and interaction and can be used to support social behaviors both between the user and the robot (e.g., companionship) and by acting as catalysts, or scaffolds, for human-human interaction. As one example of the latter, Ostrowski et al. (2019) used a participatory, mixed methods approach to study robots as tools for human connectedness in an older adult community, finding that robots prompted conversations between residents and drew them into the community space. The *Paro* robot has also been found to encourage group interaction between adults with dementia (Marti et al., 2006; Shibata and Wada, 2011).

In a systematic review, Kachouie et al. (2017) analyzed ninety-five studies investigating the use of social robots with older people and rated their outcomes against five constructs related to human well-being defined by the PERMA (positive emotion, relationships, engagement, meaning, and achievement) framework (Forgeard et al., 2011). This review found that most studies reported that social robots have the potential to improve positive emotions (such as peace, satisfaction, hope, love, security, calm). Nine studies reported an impact on relationships, including an increase in social interactions, networks, and ties (three studies), a decrease in loneliness (two studies), and facilitation of friendly interactions with peers (three studies). A more focused review of randomized control studies (Pu et al., 2018) found that social robots could improve quality of life for older adults including impacts on agitation, anxiety, engagement, stress, loneliness, and use of medications; however, meta-analysis showed a lack of robust cross-study effects. Both reviews commented on the need for additional and more rigorous studies.

One area in which social robots have demonstrated benefits is in behavior modeling, that is, in encouraging behaviors that promote well-being. A particular area where this application has proven useful for pediatric, adult, and older adult populations is in rehabilitation therapy. In this context, social robots can be used to promote engagement with self-directed exercises during (Kozyavkin et al., 2014) and between therapy sessions (Winkle et al., 2018), as well as to demonstrate specific exercises in ways that are customized for the user and the course of the treatment. Social robots can also model other types of healthy behaviors as well as activities of daily living, such as taking medication or making a cup of tea (Shishehgar et al., 2018). Social robots have been widely trialed as an intervention to scaffold social skills in children with ASD (Cabibihan et al., 2013), including training in imitation, eye contact, turn-taking and self-initiation, and learning of context appropriate social behavior.

The magnitude of benefit experienced from social robot therapeutic interventions, which integrate one or more of these dimensions, depends, in part, on attitudes and beliefs toward robots. Factors such as trust and acceptance, in combination with variables such as age, gender, culture, and prior robot exposure, are important influences in the adoption and sustained use of robot technology (Wortham and Theodorou, 2017; Langer et al., 2019; Naneva et al., 2020).

## Potential risks

The important potential benefits of social robots must be weighed against the risks they pose and evidence about the harms they could cause.

A number of commentators have argued that, because robots are designed machines, it is ethically risky, if not altogether wrong, to encourage people to treat them as social—because only other living things (principally humans and some animals) are capable of being truly social (at least for the foreseeable future). Critics include Dennett who has accused manufacturers of social robots of “false advertising” in designing robots to trigger overtly social and emotional responses in people (Dennett, 2017) (see Sharkey and Sharkey (2020) for a similar view), Sparrow and Sparrow (2002; 2006) who have described sociality in robots as intrinsically deceptive, Elder (2016) who describes relationships with robots as counterfeit, and Bryson (2010a; 2018) who has argued that forming social bonds with robots risks creating a moral obligation toward them, which goes against the best interests of human well-being.

There are a number of issues with such positions. First, other technologies, and even simple objects such as cuddly toys and dolls, are designed to elicit emotional and social engagement without undue ethical worry. Second, we are able to suspend disbelief when watching theater, TV, or film, and do not take issue with the deceptive behavior of actors in representing themselves as someone or something different to their intrinsic nature. This speaks to our sophistication as social beings and our ability to flexibly adopt different stances and to switch between them—for example, to alternately, or even simultaneously, see a robot as both an intentional agent and a designed machine or to see a robot as intentional and social, but not as having phenomenal experience or moral patiency. Third, as noted earlier, increasing evidence points to people's willingness to emotionally invest in robots, at least to some degree, and that they are already doing so with devices such as robot cleaners and pets. Social tolerance and the need to avoid stigmatization suggests that such sentiments should be respected (Danaher, 2019). Indeed, our human capacity to be concerned for things that are unable to reciprocate our concern is perhaps something to celebrate rather than to criticize (Brown, 2015).

Against the view that robots can never qualify as social entities, a relational or transactional approach would consider that what matters is not so much the category membership of robots, but the patterns and consequences of social interaction between human and robots (Coeckelbergh, 2010b; Gunkel, 2012, 2018; Danaher, 2019, 2020). This view aligns with the movement away from essentialist notions of identity (Haraway, 1991; Mischel and Shoda, 1995) and the broader relational turn in social science (e.g. Emirbayer, 1997) that sees the units (e.g. humans and robots) involved in a transaction as deriving “their meaning, significance, and identity from the (changing) functional roles they play within that transaction. The latter, seen as a dynamic, unfolding process, becomes the primary unit of analysis rather than the constituent elements themselves” (Emirbayer, 1997: p. 287). According to this systems view, inequalities, and ethical harms more broadly, derive from the unfolding relations between individuals or groups, in which essentialist attributions (for instance, stereotypes) are often part of the problem.

From this perspective then, the more pressing ethical questions concern the balance of benefits and harms that can arise from allowing robots, that people are willing to recognize as social, enter our lives. The list of potential risks and harms is still long; rather than attempt to be comprehensive, we focus here on those concerning socioemotional factors, specifically, human dignity, the potential for a reduction in, or loss of, human contact as a result of social robot use, and the broader emotional impacts of social robots.

The relationship between social robots and human dignity has been most studied in the context of robot care for older adults. At one end of the spectrum, some argue that such relationships are completely permissible, and a robot is considered as an assistive technology similar to others such as smart home systems or intelligent wheelchairs. At the other end of the spectrum, and as noted previously, some argue that social robots are inherently an affront to human dignity, as they are intrinsically deceptive and intended to replace human contact. Central to this debate and critically missing is a unifying definition of “human dignity.” Depending on context, the word dignity has been framed as a medical term, as an inherent component of human rights, and as an achievable virtue (Sharkey, 2014). Fears that social robots, for instance, as carers or companions to older adults, would reduce human dignity can be countered by evidence of mistreatment and disturbing care of older adults by fellow humans (Sharkey, 2014); in other words, there is a balance of harms to be considered. In an attempt to tackle this debate early in the social robot development process, there have been calls for the integration of human dignity as a key principle for the design and governance of social robots (Sharkey, 2014; Zardiashvili and Fosch-Villaronga, 2020).

Critics have also argued that forming relationships with robots could damage our capability to socialize with human others, for instance, by undermining our capacity for secure attachment (Sharkey and Sharkey, 2010) or our desire to engage in human-human relationships (preferring the ease, convenience, and non-challenging nature of artificial companionship) (Turkle, 2017) or by usurping our time and capacity for emotional investment (Bryson, 2018). Each of these threats deserves consideration.

Attention to how and where relationships with robots are emerging, and the extent to which they are displacing human-human relationships, is important. As noted earlier, human-robot relationships have the potential to be extremely diverse and to include forms of relationship that do not fit into any pre-existing class. The risks are likely to vary between these different settings and a clearer taxonomy and analysis of human-robot relationships building on insights from human relationship science could help. For example, the study of human relationships demonstrates that close association over a period of time can lead to deeper bonds, pointing to the possibility of greater risks (but potentially also benefits) in long-term associations with robots.

Some relationships are clearly more significant for our social development and general well-being than others. Such considerations should drive caution about the use of robots with children, for instance, where they might overlap with roles traditionally performed by primary caregivers. Nanny robots present a potential risk in this regard as highlighted by Sharkey and Sharkey (2010, 2020). On the other hand, robot dolls or pets for children can scaffold learning, promote positive behaviors such as care-giving, and provide forms of social contact that might otherwise be absent from children's lives. More broadly, worries that we exhaust our emotional capital on unfeeling artifacts, making us less able or willing to care for or befriend one another, should be set against the emerging evidence that social robots can support the acquisition of social skills, act as catalysts for forming relationships with other people, and bolster feelings of self-worth that could encourage relationship seeking.

It is worth noting the use of “slippery slope” arguments in the rhetoric surrounding some of these societal concerns. For example, worries about the use of social robots limiting access to human contact (Sparrow and Sparrow, 2006; Sharkey and Sharkey, 2012b), and the resulting psychological damage, are often predicated on supposing inappropriate and excess use of robots in, for example, child or eldercare settings, where robots could be imagined as replacing interpersonal contact largely or entirely. In order to assess such risks, we need to identify the causal chains whereby the introduction of social robots would lead to these worst-case outcomes. With respect to robot nannies, for example, Bryson (2010b), reviewing a range of risks and defeaters (such as legal liability), found the use of social robots in childcare to be “no greater danger than other artifacts and child-care practices already present in our society (Bryson, 2010b, p. 196).” This is not to dismiss the risk but to recognize that challenges such as addiction, over-dependence, and their knock-on effects on our human-human relationships are threats that social robots share with other aspects of our increasingly digitally engaged lives, from streaming services, to social media, to smartphones (Turkle, 2017). The impacts of our future relationships with robots therefore need to be considered alongside study of the broader pattern of changes to human social connectedness brought about by new technologies.

## Looking to the future

The task of gathering empirical and theoretical evidence on the role and impact of social robots can be a fairly siloed endeavor. Engineers and computer scientists develop and refine hardware and software components, advance the integration of artificial intelligence in social robots, and measure the effectiveness of human-robot interaction; among other goals. Philosophers, and other humanities scholars, explore the nature and morality of human-robot relationships in relation to broader questions about the human condition. Social psychologists, social ecologists, and relationship scientists study the dynamics of relationships, and of networks of social connection, and examine their impact on quality of life including the experience of loneliness. Technology ethicists address questions related to end-user acceptance and ethical issues such as the implications for dignity, privacy, and autonomy.

Although each of these lines of inquiry are essential to move forward, a more transdisciplinary approach, which bridges perspectives and methodologies, could allow for an in-depth understanding of relationships with social robots and their potential to improve or harm human lives. This approach could also meaningfully engage diverse stakeholders at earlier stages of prototype and product development. The potential of co-creation methods for social robotics has been demonstrated in various settings, including with children (Huijnen et al., 2017; Vallès-Peris et al., 2018) and older adults (Leong and Johnston, 2016; Lee et al., 2017; Robillard and Kabacińska, 2020). Incorporating the needs, priorities, and values of potential users, their families, and other stakeholders (e.g. care services) can address key ethical issues while increasing acceptability and adoption (Robillard et al., 2018).

**Figure 3:**
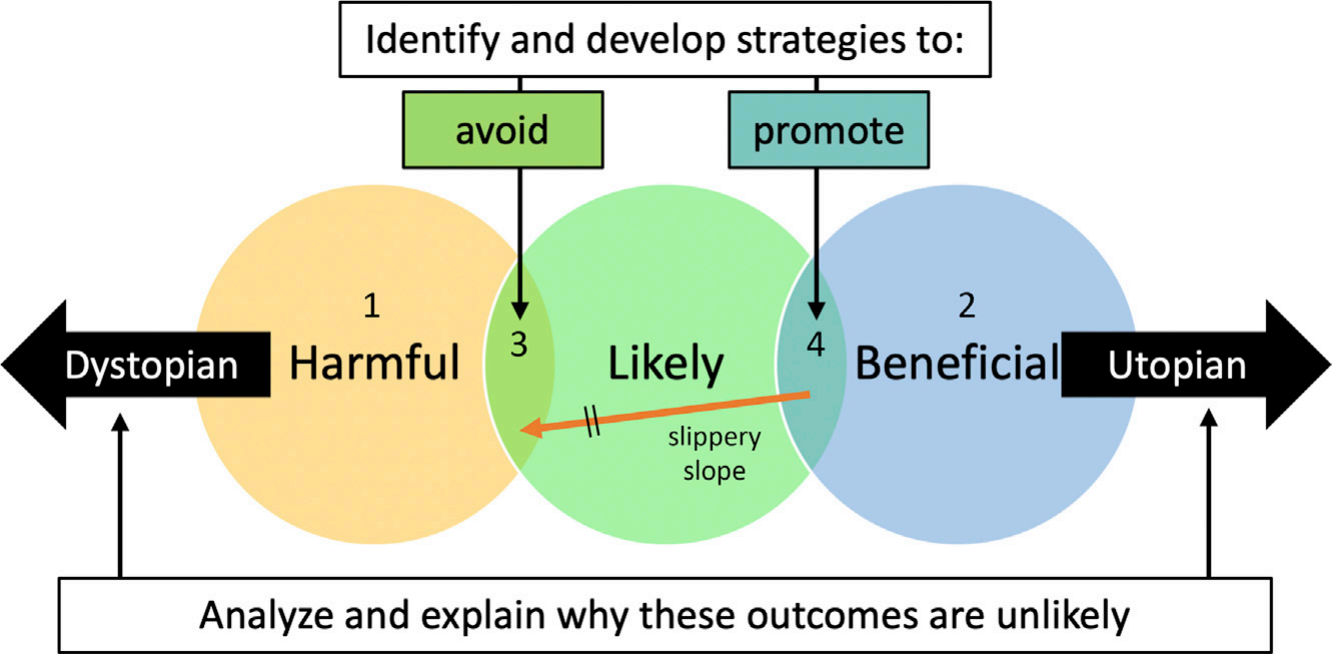
A strategy for investigating the ethical and societal impacts of social robots Consider the space of imaginable outcomes, which includes some that are harmful to human welfare (left circle) and others that are beneficial (right circle). Some outcomes are likely (central circle), others unlikely (outside the central circle). This simple scheme leads to four categories of outcomes: (1) Dystopian visions of future worlds that are very unlikely even though they are imaginable. A role for researchers is to analyze these outcomes, assess what steps would be needed for them to arise, and explain why these scenarios are unlikely to play out. (2) At the opposite extreme are the implausible, though also imaginable, utopian visions. Again, a role for researchers is to analyze and caution against unrealistic claims. Many science fiction scenarios will fall into these two categories. (3) The intersection of “harmful” and “likely” denotes negative outcomes that could happen. Having established those scenarios that are plausible we should be proactive in developing strategies that can mitigate against them. (4) Conversely, the intersection of “beneficial” and “likely” represents positive outcomes that could happen, we should develop strategies to promote these eventualities. The figure also illustrates a “slippery slope” where what might be considered to be a beneficial outcome proves to be the top of a slide into the harmful category. Slippery slopes deserve to be analyzed and we should develop safeguards (indicated by \\) against any that have a convincing causal chain (warrant). However, we should also be skeptical of slippery slopes motivated by profoundly dystopian visions, as the number of necessary steps and potential defeaters can make such scenarios highly unlikely.

The important challenges that arise when weighing the benefits and risks of human-social robot relationships translate to a lack of effective regulation and governance in this sphere. Research can play a key role in shaping both policy and practice. As illustrated in Figure 3, the challenges include to (1) consider the positive and negative potential impacts of social robots, (2) identify potential outcomes that are plausible, and (3) develop strategies to promote positive impacts and discourage negative ones. Addressing these lines of inquiry, using a transdisciplinary approach, that purposefully engages with wider society, will be critical in moving the field forward.
